# Malnutrition before kidney transplantation: how to assess it and what is the impact?

**DOI:** 10.1590/2175-8239-JBN-2023-E014en

**Published:** 2023-11-03

**Authors:** Miguel Moyses Neto, Anderson Marliere Navarro, Marcio Dantas

**Affiliations:** 1 Universidade de São Paulo, Faculdade de Medicina de Ribeirão Preto, Departamento de Clínica Médica, Divisão de Nefrologia, Ribeirão Preto, São Paulo, Brazil.; 2 Universidade de São Paulo, Faculdade de Medicina de Ribeirão Preto, Departamento de Ciências da Saúde, Divisão de Nutrição e Metabolismo, Ribeirão Preto, São Paulo, Brazil.

Compromised nutritional status is very common in patients with chronic kidney disease (CKD), especially patients on dialysis and candidates for kidney transplantation. Malnutrition in kidney transplant recipients is associated with a high rate of morbidity and mortality in the postoperative period^1^. The careful assessment of nutritional status is essential for planning behaviors that effectively meet the demands of this reality. KDIGO guidelines recommend assessment of perioperative risks for kidney transplant candidates, such as sensitization status, blood transfusions and derivatives, pregnancies, cardiovascular disease, lung diseases, infections and malignant neoplasms^2^, but there is no emphasis on nutritional assessment. In a meta-analysis with more than 50 patients with CKD, through subjective global assessment or a malnutrition-inflammation score, the researchers reported that the prevalence of protein-energy malnutrition was 28%–54% in dialysis patients^3^. However, there is a lack of reference standards and definition of cut-off points associated with clinical and nutritional risks; therefore, periodic monitoring is the best way to detect abnormalities in nutritional status, their outcomes and impacts^4^.

Santos et al.^5^ have recently proposed the use of a new nutritional assessment instrument to be applied before kidney transplantation. This instrument has the advantages of being easy to use and being of low cost, because it is based on the use of anthropometric variables, usual clinical data and routine laboratory results with the aim of evaluating the nutritional impact on clinical outcome. Although there are instruments for diagnosing malnutrition, there is no consensus on which one is the most appropriate to be applied before kidney transplantation because they all have limitations. This fact justifies the proposal to develop a new instrument or improve existing ones. Santos et al.^5^ developed and applied an assessment instrument, which was called pre-transplant malnutrition risk, to 451 patients in a retrospective cohort study. In this study, nutritional risk was obtained through the assessment of the following variables: body mass index (BMI), serum albumin levels, total cholesterol, total lymphocyte count, time on dialysis (years) and time (years) with comorbidities (diabetes mellitus and others) during the period on dialysis. From this, the patients were classified into groups of low, moderate and high nutritional risk. As a result, almost 80% of the patients were classified as moderate or high risk. Low-risk patients were younger, had a greater number of preemptive transplants, living donor transplants, and better HLA compatibility compared to groups with higher risks of malnutrition. Several variables were associated with graft loss, and this event occurred more frequently in patients in the high-risk group. These results suggest that the proposed nutritional assessment instrument can be efficient. Other positive aspects deserve recognition: the originality and easy applicability of the screening instrument; the use of information from the daily clinical practice of these patients, and the articulation of nutritional, laboratory and clinical information in a single instrument. However, improvement of the instrument is desirable. This can be achieved by incorporating functionality measures, identifying patients with frailty and inflammatory markers^6^. Certainly, the incorporation of these new variables would make the instrument by Santos et al.^5^ more complex, but it would bring the probable benefit of increasing its precision. There would also be a need for its validation by other studies and comparison with more commonly used instruments.

Some other points also deserve attention, without compromising the quality of the study. The instrument only includes BMI data, not considering the assessment of body composition and functional capacity of the muscles. It is known that this population is at greater risk of muscle mass loss, sarcopenia and cachexia, contributing to their fragility and loss of muscle functional capacity^7^. Another point that draws attention is that this instrument would be most important during the first year after the transplant. During this period, there would be a higher proportion of infections and a greater impact of immunosuppression, as well observed by the authors^5^. The same authors associated the risks of malnutrition with a decrease in graft survival, but in the medium- and long-term other factors described in this study, especially infections, depend greatly on nutritional status. Weight gain, particularly increased visceral fat, and the increased risk of developing diabetes, dyslipidemia; and cardiovascular disease, which can impact patient and graft survival^8^. Risk factors for the development of acute rejections are related to risk of sensitization, presence of donor-specific antibodies, elevated antibody panel, HLA mismatches, African American ethnicity, prolonged cold ischemia time, blood incompatibility, delayed function graft failure, non-compliance and second transplant. Episodes of graft rejection and long-term immunological changes are not directly linked to nutritional status^9^.

There is a complex interplay between overlapping nutritional and inflammatory parameters during CKD, with a need for further assessment and interpretation of these variables, and how they relate to the identification and prevention of malnutrition, specifically^10^. Therefore, as already indicated by the authors^5^, there is the possibility of new investigations improving what, in our assessment, is quality. This current study^5^ opens the way for new investigations as a way to have more validated instruments for specific purposes in the area, whether for transplant patients or not, positively impacting the intervention and ensuring better results and clinical outcomes.

## References

[1] Molnar MZ, Keszei A, Czira ME, Rudas A, Ujszaszi A, Haromszeki B (2010). Evaluation of the malnutrition-inflammation score in kidney transplant recipients. Am J Kidney Dis.

[2] Chadban SJ, Ahn C, Axelrod DA, Foster BJ, Kasiske BL, Kher V (2020). KDIGO clinical practice guideline on the evaluation and management of candidates for kidney transplantation. Transplantation.

[3] Carrero JJ, Thomas F, Nagy K, Arogundade F, Avesani CM, Chan M (2018). Global prevalence of protein-energy wasting in kidney disease: a meta-analysis of contemporary observational studies from the international society of renal nutrition and metabolism. J Ren Nutr.

[4] Ikizler TA, Burrowes JD, Byham-Gray LD, Campbell KL, Carrero JJ, Chan W (2020). KDOQI clinical practice guideline for nutrition in CKD: 2020 update. Am J Kidney Dis.

[5] Santos MRO, Lasmar MF, Nascimento E, Fabreti-Oliveira RA (2023). Impacto do risco de desnutrição pré-transplante no desfecho clínico e na sobrevida do enxerto de pacientes transplantados renais. Braz J Nephrology.

[6] Ahmadi SF, Zahmatkesh G, Streja E, Molnar MZ, Rhee CM, Kovesdy CP (2014). Body mass index and mortality in kidney transplant recipients: a systematic review and meta-analysis. Am J Nephrol.

[7] Hanna RM, Ghobry L, Wassef O, Rhee CM, Kalantar-Zadeh K (2020). A practical approach to nutrition, protein-energy wasting, sarcopenia, and cachexia in patients with chronic kidney disease. Blood Purif.

[8] Nolte Fong JV, Moore LW (2018). Nutrition trends in kidney transplant recipients: the importance of dietary monitoring and need for evidence-based recommendations. Front Med (Lausanne).

[9] Hariharan S, Israni AK, Danovitch G (2021). Long-term survival after kidney transplantation. N Engl J Med.

[10] Massini G, Caldiroli L, Molinari P, Carminati FMI, Castellano G, Vettoretti S (2023). Nutritional strategies to prevent muscle loss and sarcopenia in chronic kidney disease: what do we currently know?. Nutrients.

